# Hypoglycemia Awareness Trajectories in Young People with Type 1 Diabetes Using Flash Glucose Monitoring

**DOI:** 10.1155/2023/4882902

**Published:** 2023-10-23

**Authors:** Anissa Messaaoui, Sylvie Tenoutasse, Lucia Hajselova, Laurent Crenier

**Affiliations:** ^1^Hôpital Universitaire de Bruxelles (H.U.B), Département de Pédiatrie, Hôpital Universitaire des Enfants Reine Fabiola, Université Libre de Bruxelles (ULB), Avenue J.J Crocq 15, 1020, Bruxelles, Belgium; ^2^Hôpital Universitaire de Bruxelles (H.U.B), Département d'Endocrinologie, Hôpital Erasme, Université Libre de Bruxelles (ULB), Route de Lennik 808, 1070, Bruxelles, Belgium

## Abstract

**Aim:**

The trajectories of the hypoglycemia awareness status (HAS) have not yet been studied in children and adolescents with Type 1 diabetes (T1D).

**Methods:**

This 2-year follow-up study included children and adolescents with T1D aged 6‒20 years old and using flash glucose monitoring. The HAS of each participant was determined by the Gold score and assessed at three time points, along with clinical data. The trajectories based on HAS progression over time were identified, and a logistic regression analysis was performed to compare their characteristics.

**Results:**

Among the 255 participants, we identified four HAS trajectories (T1–T4). T1: normal awareness of hypoglycemia (NAH) maintained over time (*n* = 82, 29%); T2: NAH recovered during follow-up (*n* = 40, 18%); T3: impaired awareness of hypoglycemia (IAH) developed during follow-up (*n* = 28, 12.4%); T4: IAH maintained over time (*n* = 59, 21%). Sixteen participants (7%) displayed no identifiable trajectory. Participants belonging to the T3 group were younger. Following a specific trajectory defined the risk of developing future severe hypoglycemia.

**Conclusions:**

HAS changed in a significant proportion of pediatric people with T1D over time. Participants with a trajectory toward IAH were younger. Frequent HAS assessments may help to improve hypoglycemia risk management, especially in young children with T1D.

## 1. Introduction

Impaired awareness of hypoglycemia (IAH) is a common acquired condition in which autonomic hypoglycemia-related warning symptoms are reduced or absent in insulin-treated people with Type 1 diabetes (T1D) [1]. IAH has mainly been described in T1D, both in adults and pediatric cohorts. In children and adolescents with T1D, the IAH prevalence ranges from 20% to 40% [1, 2]. Neuroglycopenia symptoms, including cognitive impairment, may be the first manifestation of hypoglycemic episodes in patients with IAH, hence preventing or delaying corrective actions [3]. Consequently, IAH has been associated with a two to four-fold increase in the risk of severe hypoglycemia (SH) [1, 2]. For many people, the fear of experiencing SH is an obstacle to achieving therapeutic goals and indirectly increases the risk of developing chronic diabetes complications [4].

Several risk factors have been associated with IAH [1]. Indeed, recurrent hypoglycemic episodes have been shown to alter hormonal and neurogenic responses to hypoglycemia in people with T1D, even in the absence of autonomic neuropathy [5]. This phenomenon is also known as hypoglycemia-associated autonomic failure [6]. Thus, exposure to recurrent hypoglycemia may progressively impair glucose counter-regulation, reduce the ability to detect hypoglycemia in people with T1D, and lead to an increased risk of SH, creating a vicious circle [1, 7]. In children, it has been shown that the risk of recurrence of SH was the highest within the first year after an event and decreased with a longer time lag. However, this risk remains doubled up to 4 years after an event, likely partially due to a reduced sympathoadrenal response to hypoglycemia [8].

Moreover, avoidance of hypoglycemic episodes may restore a normal awareness of hypoglycemia (NAH) [9, 10]. Hypoglycemia awareness status (HAS) may, therefore, change over time [1, 2, 11], but its course seems heterogeneous and has barely been mentioned in children with T1D. In a study carried out in children and adolescents with T1D, 36% of participants reported a variation in their HAS over an 18-month period [2].

Trajectory models have been used in clinical research to understand the etiology and developmental course of various disorders [12]. Indeed, studying the longitudinal trajectories of a given clinical endpoint may help to identify different subgroups with distinct evolution patterns over time [13, 14]. For example, in a large population of persons with T1D, Schwandt et al. [15] identified five longitudinal patterns of glycemic control from childhood to early adulthood related to diabetes self-care, treatment differences, and demographics. Thus, this type of analysis enables the identification of people at risk of unfavorable glycemic control trajectories. Caregivers can then focus on preventive interventions to improve hemoglobin A1c (HbA1c) levels and reduce the risk of long-term diabetes-related complications in these individuals.

For people with T1D, HAS is closely related to the risk of experiencing SH [1]. Recognition of HAS trajectories might thus help to better assess the risk of SH over time and, as a second step, to develop specific strategies to avoid SH in at-risk people.

We, therefore, investigated whether different HAS trajectories could be identified in our population of children and adolescents using flash glucose monitoring (FGM) to manage their T1D and the clinical implications of following a given trajectory.

## 2. Methods

### 2.1. Study Design

This prospective and observational study was conducted at the Diabetology Clinic of the University Children's Hospital Queen Fabiola (Brussels, Belgium) for 2 years. Data were collected according to a standardized clinical follow-up.

### 2.2. Study Population

We enrolled people between 6 and 20 years old who had been diagnosed with T1D before 16 years of age and who were using FGM (Libre 1, without glucose threshold alarms) on a regular basis at study onset. Participants with mental disabilities or with a diabetes duration of <1 year were excluded. Any potentially eligible persons were invited to participate in this study.

Children and adolescents were treated with one of the following treatments: two daily insulin injections of an individualized mixture of rapid- and intermediate-acting insulins (Freemix Plus regimen), multiple daily injections (MDI), or insulin pump (continuous subcutaneous insulin infusion). Professional caregivers educated the young persons and their families about hypoglycemia symptoms and treatment at diagnosis and then at each follow-up visit.

### 2.3. Data Collection

Each participant's medical history, clinical data, and glucose profile were collected at baseline. IAH was assessed at baseline, at 1 year, and at 2 years by using the Gold et al. [16] method. The Gold scale is a standardized and linear Likert scale with scores ranging from 1 (the individual is always aware of hypoglycemic events) to 7 (the individual is never aware of hypoglycemic events). It has been shown to accurately identify people with T1D with IAH, both in adult [9, 10] and pediatric populations [17, 18]. In this study, children answered the Gold scale from the age of six since the literature agrees that children as young as 6 years old can give a reliable and valid self-assessment [17, 19]. We tested the Gold Scale on 100 children between 6 and 16 years old (25 children between 6 and 9 years old and 75 children between 9 and 16 years old). The evaluation was first done with the child and then by the parents. We did not find any significant differences between the answers provided by the parents and the children, even among children aged 6–9 years old. We used a cutoff score of ≥3 to diagnose IAH, as this cutoff has been validated in a pediatric population [2]. Participants with scores ≥3 were identified as having IAH; otherwise, they were considered as having a NAH status [2].

SH was defined for the purpose of this study as any hypoglycemic event leading to loss of consciousness. Episodes of SH were assessed by reviewing the logbook and were adjudicated by an endocrinologist. Nocturnal SH was defined as SH occurring during the child's sleeping hours. The events reported are those occurring during the previous year. The SH events reported are those occurring during the previous year.

FGM data were uploaded using the proprietary software (Abbott Diabetes Care, Almeda, CA, USA) at baseline, at 1 year, and at 2 years of follow-up. The results for the last 2 weeks were analyzed. Sensor-derived glycemic measures included the percentage of time spent in the target range (70–180 mg/dL), above 180 and 250 mg/dL, below 70 and 54 mg/dL, the coefficient of variation, and low blood glucose index (LBGI) [20].

Nonfasting blood samples were taken at baseline, at 1 year, and at 2 years of follow-up. HbA1c levels were measured by ion exchange, high-performance liquid chromatography (normal value <6.0% or 42 mmol/mol), and C-peptide levels by electrochemiluminescence. A random C-peptide level >0.05 nmol/L occurring in conjunction with a blood sugar level above 150 mg/dL was defined as positive.

### 2.4. Ethics

The study was approved by the hospital's medical ethical committee and performed according to the protocols used by our institution. Written informed consent was obtained from participants and from their parents when necessary.

### 2.5. Statistical Analysis

Data were reported as means ± standard deviations. Gold scores were reported as medians and interquartile ranges. We studied different possible HAS trajectories according to the “group-based trajectory modeling” concept introduced by Nagin and Odgers [12]. With three time points and only two possible HAS statuses (NAH/IAH), we were able to explore all possible trajectories without the need of a supplementary statistical methodology [12]. Comparisons between trajectories were performed using either one-way analysis of variance (ANOVA) or the *χ^2^* test. A logistic regression analysis was performed to evaluate the effects of age, gender, age at diagnosis, Gold score, C-peptide negativity, SH, number of scans, and time percentage spent below the target range, based on the likelihood that participants would follow the trajectory 3 (toward IAH). The regression results were expressed as odds ratios with 95% confidence intervals. Each variable with a *p*-value ≤ 0.1 was included in the multivariate regression model. Two-tailed statistical tests were performed using SPSS software for Windows version 28 (IBM SPSS Statistics, Chicago, IL, USA). A *p*-value < 0.05 or *p* < 0.05/*k* in the case of *k* comparisons (Bonferroni correction) was considered statistically significant.

## 3. Results

### 3.1. Participants Characteristics

Out of the 448 people aged 4‒20 years old who followed up at the clinic, 291 met the study's inclusion criteria. Subjects excluded were not FGM users (*n* = 111; 35 were using another glucose sensor, and 76 were using capillary blood glucose tests), had a mental disability (*n* = 7), or had a diabetes duration <1 year (*n* = 39). Eight of them refused to participate, and the 283 remaining participants were enrolled (Supplementary 1). None of the people enrolled were on sensor-augmented pumps or automated insulin delivery. The mean follow-up duration was 2.0 ± 0.1 years. At baseline, 134 participants (47%) were diagnosed with IAH. Twenty-two percent of participants diagnosed with IAH experienced at least one SH event, as opposed to 7% of NAH participants (*p* < 0.001). Participants with IAH also displayed nocturnal SH more often (10% in IAH vs. 3% in NAH, *p*=0.013). They had a higher time percentage spent below the target range (16% ± 9% in IAH vs. 13% ± 7% in NAH, *p*=0.002) and a higher time percentage spent below 54 mg/dL (7% ± 3% in IAH vs. 6% ± 4% in NAH, *p*=0.002) compared with those with NAH. There were no significant differences regarding age, diabetes duration, insulin regimen, or HbA1c levels.

### 3.2. Drop-Out

Fifty-eight participants were lost to follow-up: 28 with IAH and 30 with NAH. These were older (*p* < 0.001), older at diabetes diagnosis (*p* < 0.001), and had a longer diabetes duration (*p* < 0.001) than participants who participated until the end of the study. There were no notable differences in terms of HAS (Supplementary 2).

### 3.3. Trajectory Groups

**Figure 1:**
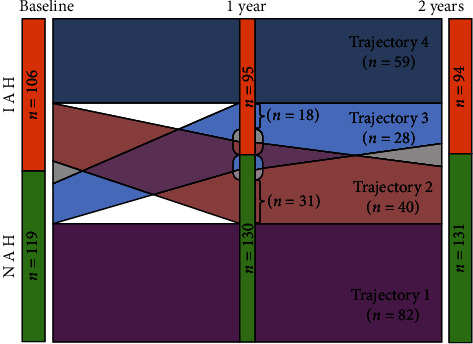
Impaired awareness of hypoglycemia trajectories, evolution at baseline, at 1 year, and at 2 years of follow-up.

We identified four HAS trajectories (Figure 1): trajectory 1 was the most frequent and included participants maintaining NAH over time (*n* = 82, 29%). Trajectory 2 included those who had IAH at baseline and reverted to NAH (*n* = 40, 18%). Trajectory 3 included participants who had NAH at baseline and developed IAH during follow-up (*n* = 28, 12.4%). Trajectory 4 gathered participants who maintained their IAH status over time (*n* = 59, 21%). Sixteen participants (7%) displayed no identifiable IAH trajectory.

Participants following trajectory 3, who developed IAH during follow-up, were younger than those whose status did not change (Table 1). In trajectory 1 (always NAH), we found a lower time percentage spent below the target range, a lower time percentage spent below 54 mg/dL, and a lower LBGI (Table 1). We did not find any difference in diabetes duration, HbA1c levels, or gender. As expected, there were no significant differences regarding the Gold scores of trajectories 1 and 4 at each time point.

For all four trajectories, the HAS at baseline and at 1 year were directly related to the risk of developing SH during the following year; thus, for each observation period, there were significantly more SH episodes in the year following the IAH diagnosis, and when the participant reverted to NAH, the risk of future SH was decreased (Table 2). On the contrary, NAH recovery reduced the risk of hypoglycemia, and belonging to trajectory 2 significantly reduced the risk of future SH.

In the multivariate analyses, the SH occurrence (odds ratio (OR) 2.8 (1.8, 5.0); *p*=0.039), baseline Gold score (OR 2.4 (1.6, 3.6); *p* < 0.001), and C-peptide negativity (OR 3.5 (1.2, 10.7); *p*=0.042) were predictive factors of trajectory 3, toward IAH (Table 3).

IAH frequency remained relatively stable over time: 47% at baseline, 48% after 1 year, and 48% after 2 years (*p*-value nonsignificant).

## 4. Discussion

This prospective and observational study implemented a systematic description of HAS trajectories over 2 years in a cohort of children and adolescents with T1D using FGM. The HAS was determined by the Gold score, a tool recently validated in a pediatric population [2]. This method is also remarkably simple to use in routine clinical practice [2]. Since more than 97% of eligible persons agreed to participate, this study was representative of the HAS trajectories among young people with T1D followed-up in our clinic. IAH prevalence at each time point was in line with other pediatric studies [2, 21]. However, the use of the sensor was not widespread in these studies, making rigorous comparisons difficult.

We were able to demonstrate the existence of four HAS trajectories in our children and adolescents with T1D using FGM. While the overall IAH frequency remained relatively consistent over the 2-year follow-up, this global finding masks very different clinical histories between individuals.

The proportion of participants experiencing episodes of SH was highest among those following trajectory 4 (always IAH). Moreover, SH occurrence was higher during the year following the IAH diagnosis, and similarly, the risk of future SH decreased after reversion to NAH. This validated the use of the Gold et al. [16] scale and confirmed, in children, the well-demonstrated link between HAS and SH risk in adults. The known reversible factors for developing hypoglycemia-associated autonomic failure and IAH are frequent hypoglycemia episodes, sleep, or prior exercise [22]. In adults, other risk factors include long-standing diabetes [23, 24], C-peptide negativity [22], and possible genetic factors [25]. Although we confirmed that a negative C-peptide is a risk factor for developing IAH in children, our findings also support other mechanisms in our pediatric population. Indeed, 25% of our participants were still C-peptide positive at IAH diagnosis, while conversely, C-peptide negativity is present in most adults with IAH [22].

In adults with T1D, the presence of nonreversible risk factors, such as long-standing diabetes or beta-cell failure, suggests that IAH may typically persist for years [26]. In contrast, HAS appeared to follow different trajectories over a short period of time in a significant proportion of our pediatric people.

In this study, we found that participants who followed either trajectories 2 or 3 (with HAS modification) were younger. Hypoglycemia symptoms in young children with T1D clearly differ from those experienced by adults and include behavioral changes such as the primary features of low blood glucose [23]. The dynamic process leading to hypoglycemia recognition by children with T1D has already been analyzed by Graveling et al. [7]. The authors proposed the concept of a “voluntary” component to hypoglycemia awareness: the ability to recognize hypoglycemia can improve with better physical and mental maturation, allowing for better hypoglycemia management by the child and his/her family.

In this study, we used a threshold of 3 for the diagnosis of IAH. In adults, the threshold generally used is 4 [1], but Hatle et al. [2] recently demonstrated that a threshold of 3 allowed better discrimination in pediatrics. Admittedly, using 3 instead of 4 may possibly overestimate the proportion of people with IAH, but it has been shown that using the threshold of 3 in pediatrics allowed better identification of people at risk of developing SH [2]. Furthermore, in our population, by using a threshold of 4 (data not shown) has enabled us to highlight the existence of status trajectories in proportions similar to those presented in the present study. However, as shown in Hatle's article, using a threshold of 4 was less discriminating for HS than using a threshold of 3.

During the follow-up period, 58 participants were lost to follow-up. Their characteristics did not differ significantly from those of the other participants except for an older age and for the insulin regimen. This is mainly because the study was conducted in a pediatric diabetes center, and patients from the age of 16 can choose to join a center for adults. The switch to MDI occurs in our oldest patients, who are close to the transition to adult care. In any case, we did not show any impact of the insulin regimen on HAS trajectories.

Our study has several strengths but also some limitations. To our knowledge, we reported for the first time different HAS trajectories in a pediatric population using FGM for diabetes management. As the observational design allowed us to include most children and adolescents followed up in our center, the current study avoids several confounding effects of interventional studies (e.g., people with IAH more willing to participate, etc.). Moreover, the systematic use of the FGM among participants prevented other possible biases due to different monitoring methods and a better glucose data capture. On the other hand, the observational nature of this work does not allow us to establish causal links. Another limitation could be the low number of SH reported. Nevertheless, despite this low number, we demonstrated a significant link between SH events and the HAS. Since this is a single-center study, it may also not be fully representative of other populations. Another weakness could be the relatively short evaluation period.

In conclusion, this study showed that a significant proportion of pediatric people with T1D may either revert to NAH or develop IAH in a relatively short period of time, following different HAS trajectories. Participants with a trajectory towards IAH were younger. Frequent HAS assessments may, therefore, help to improve hypoglycemia risk management in young people with T1D.

## Figures and Tables

**Table 1:** Baseline characteristics of the study population according to the four trajectories.

	Overall population *n* = 225	Trajectory 1 always NAH *n* = 82	Trajectory 2 → NAH *n* = 40	Trajectory 3 → IAH *n* = 28	Trajectory 4 always IAH *n* = 59	*p*-Value tot
Baseline gold score	2 (1–4)	1 (1–2)	4 (3–4)	2 (1–2)	4 (3–4)	<**0.001**
Age (years)	12.2 ± 3.2	13.1 ± 2.7	11.5 ± 3.1	10.6 ± 3.3	12.5 ± 3.2	<**0.001**
Age <9 years, *n* (%)	42 (19)	6 (7)	11 (28)	9 (32)	11 (19)	0.003
Male gender, *n* (%)	110 (49)	42 (51)	18 (45)	15 (54)	32 (54)	0.925
Age at diagnosis (years)	7.0 ± 3.6	7.7 ± 3.7	6.2 ± 3.1	6.7 ± 3.2	6.9 ± 3.6	0.192
Diabetes duration (years)	5.1 ± 3.2	5.3 ± 3.4	5.2 ± 2.6	3.8 ± 2.7	5.5 ± 3.5	0.205
Insulin regimen, *n* (%)						0.187
Freemix plus	187 (83)	67 (82)	31 (78)	24 (85)	52 (88)	0.530
MDI	17 (8)	10 (12)	3 (6)	1 (3)	3 (5)	0.246
CSII	21 (9)	5 (6)	6 (16)	3 (11)	4 (7)	0.160
C-peptide negative, *n* (%)	151 (67)	44 (54)	22 (55)	21 (75)	39 (66)	0.146
HbA1c (%)	7.7 ± 1.2	8.0 ± 1.3	7.7 ± 1.0	7.7 ± 1.2	7.6 ± 1.1	0.159
SH, *n*	0.2 ± 0.5	0.1 ± 0.2	0.2 ± 0.5	0.1 ± 0.3	0.4 ± 0.9	**0.004**
SH/participant, *n* (%)						0.029
No event	197 (88)	77 (94)	33 (83)	26 (93)	46 (78)	0.036
One event	19 (8)	5 (6)	5 (13)	4 (14)	6 (10)	0.801
More than one event	10 (4)	0	2 (5)	1 (4)	7 (12)	**0.001**
Nocturnal event	12 (5)	3 (4)	3 (8)	0	6 (10)	0.132
Scans, *n*/day	7.5 ± 4.5	7.3 ± 4.5	8.4 ± 4.6	6.6 ± 3.2	7.7 ± 5.1	0.601
Time spent in target range (%)	41 ± 13	41 ± 14	43 ± 15	42 ± 12	41 ± 12	0.883
Time spent below 70 mg/dL (%)	14 ± 8	12 ± 7	13 ± 6	15 ± 7	17 ± 10	<**0.001**
Time spent below 54 mg/dL (%)	6 ± 5	5 ± 4	6 ± 4	7 ± 5	8 ± 5	**0.002**
Coefficient of variation (%)	52 ± 8	51 ± 8	53 ± 8	53 ± 7	54 ± 8	0.172
Low blood glucose index	3.9 ± 3.1	3.1 ± 1.8	4.1 ± 3.3	3.8 ± 2.0	4.9 ± 2.9	**0.015**

All values are shown as mean ± SD excluding Gold score as median (IQR) and gender, insulin regimen, C-peptide negativity, and participants with SH as *n* (%). Comparisons between groups were performed using one-way ANOVA or *χ^2^* test or Fisher's exact test (gender, insulin regimen, C-peptide negativity, IAH, severe SH/participant) depending on the subgroup size. The threshold for significance of comparisons within each panel was *p* < 0.05/15 or *p* < 0.003. Abbreviations: MDI, multiple daily injections; CSII, continuous subcutaneous insulin infusion; HbA1c, hemoglobin A1c; IAH, impaired awareness of hypoglycemia; IQR, interquartile range; NAH, normal awareness of hypoglycemia; SD, standard deviation; SH, severe hypoglycemia. Results in bold values are considered as statistically significant.

**Table 2:** Severe hypoglycemia episodes during the first and second year of follow-up.

	IAH at baseline (*n* = 106)	NAH at baseline (*n* = 119)	*p*-Value
*First year*			
SH/participant, *n* (%)			
No event	85 (80)	107 (90)	**0.031**
One event	15 (14)	8 (7)	**0.033**
More than one event	6 (6)	4 (3)	0.304
Nocturnal event	11 (10)	6 (5)	0.066
*Second year*			
SH/participant, *n* (%)			
No event	93 (88)	110 (92)	0.267
1 event	9 (9)	7 (6)	0.604
More than 1 event	4 (4)	2 (2)	0.423
Nocturnal event	4 (4)	3 (3)	0.709

	IAH after 1 year (*n* = 95)	NAH after 1 year (*n* = 130)	*p*-Value

*Second year*			
SH/participant, *n* (%)			
No event	79 (83)	124 (95)	**0.003**
One event	11 (12)	5 (4)	**0.035**
More than one event	5 (5)	1 (1)	0.086
Nocturnal event	6 (6)	1 (1)	0.024

Comparisons between participants with IAH and NAH (*n* = 225). All values are shown as *n* (%). Comparisons between groups were performed using *χ*^2^ test, with Yates' correction or Fisher's exact test. Abbreviations: IAH, impaired awareness of hypoglycemia; NAH, normal awareness of hypoglycemia; SH, severe hypoglycemia. Results in bold values are considered as statistically significant.

**Table 3:** Adjusted logistic regression analysis of trajectory 3 (toward IAH) predictors.

	Univariate analysis		Multivariate analysis^†^	
	Odds ratio (95% CI)	*p*-Value	Odds ratio (95% CI)	*p*-Value
Age	0.920 (0.827, 1.024)	0.126		
Gender (male)	0.827 (0.416, 1.643)	0.588		
Age at diagnosis	0.927 (0.838, 1.025)	0.137		
Gold score	2.252 (1.640, 3.091)	<**0.001**	2.432 (1.654, 3.575)	<**0.001**
Insulin regimen	5.333 (0.257, 11.797)	0.279		
Diabetes duration	1.008 (0.906, 1.122)	0.884		
HbA1c level	0.918 (0.681, 1.237)	0.574		
C-peptide negativity	3.200 (1.174, 8.725)	**0.023**	3.538 (1.170, 10.700)	**0.042**
Severe hypoglycemia	2.657 (1.651, 4.214)	**0.022**	2.815 (1.750, 4.982)	**0.039**
Number of scans	1.041 (0.968, 1.118)	0.277		
Percentage of time spent in the target range	1.013 (0.985, 1.042)	0.372		
Percentage of time spent below 70 mg/dL	1.004 (0.955, 1.055)	0.881		
Percentage of time spent below 54 mg/dL	0.997 (0.941, 1.057)	0.927		
Coefficient of variation	0.989 (0.948, 1.031)	0.589		
LBGI	1.018 (0.873, 1.186)	0.825		

^†^Binomial logistic regression model: *χ*^2^ = 52.662, df = 3, *p* < 0.001; Nagelkerke *R*^2^ = 39.3%; correctly classified cases = 83%. Abbreviations: CI, confidence interval; HbA1c, hemoglobin A1c; LBGI, low blood glucose index; IAH, impaired awareness of hypoglycemia. Results in bold values are considered as statistically significant.

## Data Availability

Data supporting this research article are available from the corresponding author upon reasonable request.

## Supplementary Materials

Supplement 1 Flowchart of the study.

Supplement 2 Characteristics of the study population: lost to follow-up vs. complete follow-up. (Cryer PE. Mechanisms of hypoglycemia associated autonomic failure in diabetes. N Engl J Med 2013; 369(4): 362–372).
