# Supplementary material for: Hypoglycemia Awareness Trajectories in Young People with Type 1 Diabetes Using Flash Glucose Monitoring
Source: Pediatr Diabetes. 2023 Oct 23;2023:4882902. doi: 10.1155/2023/4882902 (PMC12016714; doi:10.1155/2023/4882902)
Supplement: Supplement 1 — Flowchart of the study. [file 4882902.f1.docx]

Supplementary figure 1–Flow chart of the study

**All patients aged 6-20 years, with Type 1 diabetes followed-up at the clinic**

**(n=448)**

**Excluded subjects (n=157)**

**Reasons:**

- **Diabetes duration <1 year, n=39**
- **Mental disability, n=7**
- **No Flash Glucose Monitoring, n=111**

**All eligible participants**

**(n=291)**

**IAH questionnaire**

**(n=283)**

**No answer to the IAH questionnaire**

**(n=8)**

**IAH *i.e.,* score ≥3**

**(n=134; 47%)**

**NAH *i.e.,* score <3**

**(n=149; 53%)**

Abbreviations: IAH, impaired awareness of hypoglycemia; NAH, normal awareness of hypoglycemia
