# Supplementary material for: Hypoglycemia Awareness Trajectories in Young People with Type 1 Diabetes Using Flash Glucose Monitoring
Source: Pediatr Diabetes. 2023 Oct 23;2023:4882902. doi: 10.1155/2023/4882902 (PMC12016714; doi:10.1155/2023/4882902)
Supplement: Supplement 2 — Characteristics of the study population: lost to follow-up vs. complete follow-up. (Cryer PE. Mechanisms of hypoglycemia associated autonomic failure in diabetes. N Engl J Med 2013; 369(4): 362–372). [file 4882902.f2.docx]

Supplementary table 1–Characteristics of the study population: lost to follow-up *vs.* complete follow-up

|  | Drop out  n=58 | At FUP  n=225 | P-value |
| --- | --- | --- | --- |
| Age, years | 17.5±2.2 | 12.2±3.2 | **<0.001** |
| Male, n (%) | 32 (55) | 110 (49) | 0.462 |
| Age at diagnosis, years | 9.9±3.9 | 7.0±3.6 | **<0.001** |
| Diabetes duration, years | 7.5±3.9 | 5.1±3.2 | **<0.001** |
| Insulin schema, n (%)  -Freemix Plus  -MDI  -CSII | 30 (52)  26 (45)  2 (3) | 187 (83)  17 (7)  21 (10) | **<0.001**  **<0.001**  **<0.001**  0.266 |
| HbA1c, % | 7.9±1.1 | 7.8±1.2 | 0.577 |
| C-peptide negative, n (%) | 37 (73) | 142 (63) | 0.504 |
| Gold score | 2 (1–3) | 2 (1–4) | 0.483 |
| IAH (gold score ≥3), n (%) | 28 (48) | 106 (47) | 0.884 |
| SH/participant, n (%)  - No event  - One event  - More than one event  - Nocturnal event | 47 (81)  8 (14)  3 (5)  6 (10) | 197 (88)  19 (8)  9 (4)  12 (5) | 0.404  0.204  0.315  0.716  0.222 |
| Scans, n/day | 6.6±7.1 | 7.5±4.5 | 0.232 |
| Time spent in the target range, % | 42±11 | 41±13 | 0.907 |
| Time spent above 180 mg/dl, % | 44±15 | 44±16 | 0.915 |
| Time spent above 250 mg/dl, % | 24±14 | 25±14 | 0.631 |
| Time spent below 70 mg/dl, % | 14±8 | 14±8 | 0.897 |
| Time spent below 54 mg/dl, % | 7±5 | 6±5 | 0.647 |
| Coefficient of variation, % | 52±9 | 52±8 | 0.943 |
| Low Blood Glucose Index | 4.0±3.0 | 3.9±3.3 | 0.827 |

- All values are shown as mean ± SD excluding gender, insulin schema, C-peptide negativity, IAH, SH/participant as n (%) and Gold score as median (IQR)
- Abbreviations: FUP, follow-up; MDI, multiple daily injections; CSII, Continuous Subcutaneous Insulin Infusion; HbA1c, hemoglobin A1c; IAH, impaired awareness of hypoglycemia; IQR, interquartile range; SD, standard derivation; SH, severe hypoglycemia.
- Comparisons between groups were performed using independent samples *t* test or Chi-square test or Fisher’s exact test (gender, insulin schema, C-peptide negativity, IAH, severe SH/participant) depending on the subgroup size.
- The threshold for significance of comparisons within each panel was p<0.05/18 or p<0.003.
